# Microplastic Pollution in Agricultural Waters in the Mississippi Delta

**DOI:** 10.3390/jox16030081

**Published:** 2026-05-06

**Authors:** Edward Heinen, James V. Cizdziel, Boluwatife S. Olubusoye, Ruojia Li, Matthew T. Moore, Lindsey M. Witthaus

**Affiliations:** 1 Department of Chemistry and Biochemistry, University of Mississippi, University, Oxford, MS 38677, USA; 2 Water Quality and Ecology Research Unit, U.S. Department of Agriculture, Agricultural Research Service, Oxford, MS 38655, USA; matt.moore@usda.gov (M.T.M.);

**Keywords:** microplastics, plasticulture, water pollution, agricultural pollution, Mississippi Delta, µ-FTIR

## Abstract

Microplastic (MP) pollution in agricultural settings is an emerging field of study, with interest focusing on potential resource contamination of soil and water due to the use of plastic materials in farming practices. The Mississippi Delta, a highly agricultural region, is prone to both natural and intentional flooding, potentially exacerbating this issue. This exploratory study investigated MP (>30 µm) concentrations, sizes, and polymer compositions in floodwater, irrigation water, and surface runoff from soybean fields across two counties in the Mississippi Delta using micro-Fourier transform infrared spectroscopy (µ-FTIR). Mean ± SE concentrations (MPs/L) were 72 ± 66 in floodwater (*n* = 18), 169 ± 121 in source (irrigation pond) water (*n* = 4), and 30 ± 37 in runoff (*n* = 3) in Sunflower County, MS. In Coahoma County, MS, mean ± SE runoff concentration was 88 ± 76 MPs/L (*n* = 24). Mean concentrations were elevated as compared to other MP studies in agricultural environments. The most common polymers present were polyethylene terephthalate (PET), polyethylene (PE), polyethylene-ethyl vinyl acetate (PE/EVA), and thermoplastic elastomers (TPEs), which are commonly used in the manufacturing of agricultural materials. MPs from the smallest size fraction measured (30–100 µm) were the most common in all floodwater samples, ranging from 75.5–91% abundance. Using Attenuated Total Reflectance (ATR)-FTIR, larger plastic litter was identified as mostly PE and PET, which is consistent with polymer distributions in floodwater samples. Overall, MPs were prevalent in both floodwater and runoff, with relatively consistent concentrations and polymer compositions across samples. However, further research is needed to fully elucidate their fate and potential impacts on agricultural systems.

## 1. Introduction

### 1.1. Plasticulture

Agriculture is the nexus of human and environmental health concerns, as it represents the largest interface between humans and the environment [1]. Plasticulture is the use of plastic materials for agricultural applications [2]. In agriculture, plastic materials are often used for mulching, irrigation tubing, greenhouses, and shade films [3,4,5]. The most used polymers in agriculture include polyethylene (PE), polypropylene (PP), polystyrene (PS), EVA (ethyl vinyl acetate), polyvinyl chloride (PVC), polyethylene terephthalate (PET), polycarbonate (PC), poly (methyl methacrylate) (PMMA), thermoplastic polyurethane (TPU), polyamide (PA), and acrylonitrile butadiene styrene (ABS) [6].

Plastics are typically categorized by size as follows: nanoplastics (1–<1000 nm), microplastics (1–<1000 µm), mesoplastics (1–<10 mm), and macroplastics (≥1 cm) [7,8]. In agricultural fields, high exposure to sunlight is a major source of polymer degradation of larger plastics into MPs [7]. Other common sources of environmental plastic degradation include abiotic pathways like mechanical and chemical degradation, while biotic pathways include microbial degradation [9]. As a result, MPs have been found in agricultural soils, irrigation water, and runoff [10,11]. Agricultural fields not only serve as sinks for MPs embedded deep in the soil, but also as sources when soil MPs in the surface layer are carried away in runoff. However, further studies are needed to assess the full extent of MP pollution in agriculture. Given the potential human health and environmental risks of MPs, it is crucial to determine not only the amounts and types of MPs present in these environments, but also the transport and fate of agricultural MPs.

### 1.2. Microplastic Transport and Fate in Agricultural Environments

Vertical transport of MPs in agricultural soils is facilitated by harvesting and tilling, and soil organisms such as earthworms can accumulate MPs on their exterior and transport them deeper into the soil [12,13]. Due to their small size and light weight, MPs near the surface are easily transported in moving water, even in currents as weak as agricultural runoff [14,15]. Lateral movement of MPs in topsoil occurs when large amounts of rainfall erode soils, breaking loose MPs [16]. Rainfall also acts a potential source of MPs, and some studies have noted concentrations that make it a significant source of environmental MP transport [17]. Runoff that is not recycled for irrigation may enter larger streams, rivers, and lakes, thereby exposing wildlife to MPs and their potential harmful effects. In addition to direct precipitation atmospheric deposition of MPs by wind is another concern [18]. In this transport route, MP fragments and fibers tend to come from local and regional sources ranging 10–100 km [18]. The presence and density of vegetation in fields helps to minimize the lateral transport of MPs in runoff by providing a physical barrier, and MPs are more likely to be transported in runoff on fallow fields [14]. Additionally, MPs in fields with crops have led to negative foliar interactions that interfere with plant metabolism, photosynthetic impairment, and oxidative damage [19,20].

### 1.3. The Mississippi Delta and Flooded Agricultural Fields

The Mississippi Delta is a region along the northwest border of the state with a long history of intensive agricultural activity [21]. Positioned along the Mississippi River, the Delta contains approximately 4.2 million hectares of agricultural land that produce soybeans, corn, rice, and cotton [22].

Corn and soybean fields in the lower Mississippi Delta have been flooded post-harvest with surface water (tailwater recovery ditches and ponds) to study the impact of shallow water on soil nutrients in the fall and winter, as well as to provide habitat for migratory shorebirds [23]. Fall and winter flooding decreased CO_2_ efflux and significantly increased soil pH and residue decomposition [23]. It is important to consider how this floodwater may influence MP concentrations in these types of post-harvest, shallow flooded fields.

### 1.4. Vibrational Spectroscopy for Plastic Identification

Raman spectroscopy, pyrolysis-gas chromatography-mass spectrometry (Pyr-GC-MS), and Fourier transform infrared spectroscopy (FTIR) are the most common methods used to identify MPs [24]. However, since Pyr-GC-MS is a destructive technique, vibrational spectroscopy is preferred to preserve samples for further analyses [24]. Raman spectroscopy is subject to fluorescent interference, and the diffraction limit of the laser limits imaging of smaller MPs [25]. As a result, the leading spectroscopic techniques are attenuated total reflectance (ATR)-FTIR and µ-FTIR, which are used for large plastic materials and MPs, respectively [24]. Micro-FTIR combines the power of microscopy with spectroscopy, leading to high-resolution images of the MPs used when recording point-specific, spectral data. ATR-FTIR is useful for larger materials, and both methods of FTIR have high throughput, require a small sample amount, and are efficient at analyzing environmental samples [24].

### 1.5. Study Background

This study examined MP pollution in agricultural fields in the Mississippi Delta for the first time. Specifically, µ-FTIR was used to identify and quantify MPs in post-harvest flooded fields, as well as in source water and runoff. At the post-harvest study site, shallow flooding was carried out by pumping recycled irrigation water from a tailwater recovery pond (TWRP) through PE piping. An initial comparison was made to determine the difference in MP concentrations, polymer types, and size distributions between two post-harvest flooded fields. Following this, one of the fields was selected for further sampling, and the source water and drainage ditch were also sampled. This study was intended to examine the concentrations of MPs in floodwater, as well as how the fields may serve as a sink for MPs from contaminated water and a source of MPs in runoff. Pieces of plastic litter including bottles were collected within and on the perimeter of the field to provide an initial determination of where MPs may have originated, given the challenges of specific source identification.

The second sampling site did not use fall and winter flooding, but fields were irrigated during the cropping season using PE piping. In the event of heavy rainfall, end of field runoff risks flushing MPs off the field, contaminating nearby waterways. Samples were collected from this site during rain events in order to identify and characterize MPs found in stormwater runoff.

By providing preliminary data on MP concentrations and polymer types at these agricultural fields, we hope to lay the foundation for future research into how MPs may be present in irrigation, standing flood and runoff waters, as well as how MPs affect migratory shorebirds.

## 2. Materials and Methods

### 2.1. Study Site and Sampling

Duplicate grab samples of standing floodwater were collected from two post-harvest flooded fields (A and B), each 8 ha, in Sunflower County, MS (near Indianola, MS) on 14 November 2023. Both fields had been planted in soybeans. Field A was flooded for the fall only (September–November), while Field B was flooded for the fall and winter (September–January). Samples (A, *n* = 6; B, *n* = 6) were collected in one quart (0.95 L) glass Mason jars. Careful efforts were made to avoid agitating soil that would contaminate water samples. Various potential sources of MPs were also collected, including small pieces of plastic irrigation piping, mulching films, tarps, and plastic litter. To ensure sample integrity, collection was performed in stagnant areas where water remained undisturbed by movement in adjacent rows. The path to the sampling point was taken in a neighboring row, and the sampling row was left undisturbed. During processing, none of the samples contained enough suspended soil to interfere with any steps nor the overall analysis. All samples were sealed and returned to the laboratory, and water samples were placed in a refrigerator at 4 °C until ready for sample analysis.

In Fall 2024, the tailwater recovery pond (TWRP) (*n* = 2) and tailwater recovery ditch (TWRD) (*n* = 1) were sampled on 8 September 2024, prior to post-harvest flooding of the fields. On 17 November 2024, floodwater samples were collected from field A (*n* = 6) after it was flooded, and TWRP (*n* = 2) and TWRD (*n* = 2) samples were collected as before during the previous sampling. Grab samples were collected using the same procedure from Fall 2023.

In addition to the flooded fields, two fields (C, *n* = 12, 67 ha and D, *n* = 12, 36 ha) in Coahoma County, MS (near Clarksdale, MS), were sampled during rain events from September 2024 to February 2025. Both fields were planted with soybeans for the 2024 season. These fields do not undergo annual post-harvest flooding as in sites A and B. All samples were collected from a steel outflow pipe draining into a ditch at the side of the fields. An automatic sampler was deployed to catch runoff, and samples were collected in the same pre-cleaned, 0.95 L glass Mason jars.

Note: GPS coordinates are not included at the request of the farm owners, who wished to remain unidentified.

### 2.2. Sample Preparation

**Figure 1:**
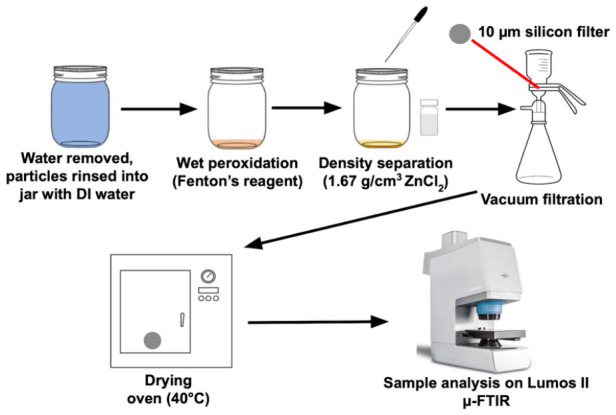
One-pot sample preparation flow diagram for MP analysis of water samples.

To minimize contamination and loss of MPs, water samples were processed in their original container using a “one pot” method (Figure 1) [26,27]. A 30 µm Monel screen (Unique Wire Weaving Co., Hillside, NJ, USA) was placed over the open mouth of Mason jars, and lid rings were replaced prior to pouring the water. Use of this screen limited results of this study to particles > 30 µm. Remaining particles on the screen were rinsed back into the sampling jar using MilliQ^®^ water (MilliPore Sigma, Burlington, MA, USA). Organic matter was digested using Fenton’s reagent (2:1 30% H_2_O_2_:0.05 M Fe(II)), followed by a density separation in ZnCl_2_ (1.6 g/cm^3^). While highly effective for digesting organic matter, it is important to note that Fenton’s reagent has shown the ability to slightly alter surface area and overall mass of MPs, with a preference for those with aromatic rings [28]. Density separation using ZnCl_2_ was selected because the densities of most plastic polymers are below 1.6 g/cm^3^ and will thus float to the surface for removal [29,30]. Additionally, this solution is cost-effective, as it is cheaper than NaI and can be reused up to 5 times with >95% recovery efficiency [29].

A glass pipet was used to transfer the top layer of solution to a glass scintillation vial. After the top layer was removed, the jar was gently shaken, left to sit for approximately 4 h, and then the top layer was pipetted into the same vial; the entire process was repeated a total of three times. Once MPs were isolated in this solution, they were vacuum filtered onto a clean 20 mm diameter silicon filter with 13 µm pores for analysis. Silicon filters are transparent in the mid-IR range, which allowed IR light to interact with the MPs in transmission mode.

**Figure A1:**
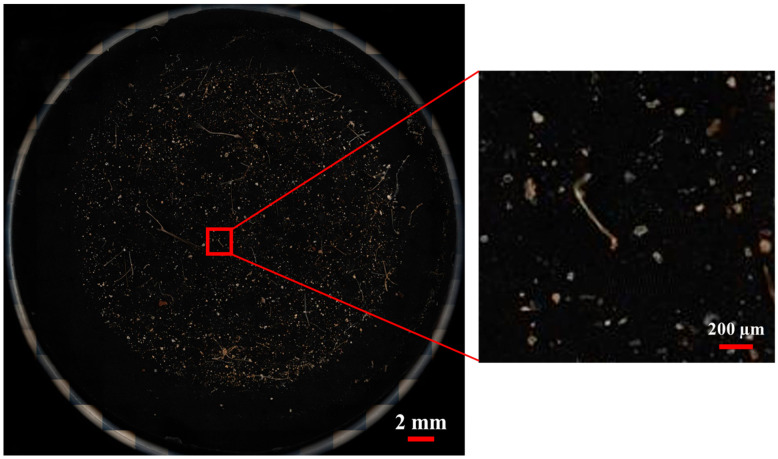
Microscopic (optical) image of all particles filtered from an agricultural floodwater sample onto a silicon filter, including microplastics, sediment, and other particles, along with a zoomed-in image showing fragments and a fiber.

An optical image of a silicon filter showing the particles (with a zoomed-in area) for a representative sample is provided in Figure A1. Note that particle morphology was not assessed or quantified in this exploratory study.

All samples were processed in a clean room while wearing cotton lab coats and nitrile gloves. Glassware was cleaned using MilliQ^®^ water filtered through a 0.22 µm filter, followed by heat cleaning at 450 °C for 4 h.

### 2.3. Polymer Identification Using µ-FTIR in Transmission Mode for Individual MPs and ATR-FTIR for Macroplastics

A Bruker LUMOS II µ-FTIR with a mercury cadmium telluride (MCT) detector cooled by liquid nitrogen was used to record spectra of the MP particles on the filter (Bruker Corp., Billeraca, MA, USA). The instrument automatically locates particles on the filter via its particle-find feature and performs individual spectral analysis on each particle. Background and sample spectra were recorded in transmission mode from 4000 to 650 cm^−1^ with 4 cm^−1^ resolution and 16 replicate scans for µ-FTIR samples. This setup is optimized for analysis of MPs in transmission mode on silicon filters.

For a few pieces of plastic litter (macroplastics) found in the agricultural fields, the same instrument was used but in conventional ATR-FTIR mode. Here, the plastic piece was placed on a diamond ATR crystal and lightly pressed to make good contact with the crystal surface. IR spectra were recorded using the same parameters listed above with the exception of using 64 replicate scans instead of 16 scans.

Spectral data were processed using OPUS 8.7.4 software, and the Cluster ID function was used to match spectra to polymer libraries. Bruker’s polymer libraries, as well as unweathered and environmental FTIR libraries of plastic particles (FLOPP and FLOPP-e) were used to match spectral data to polymer IDs [31]. Any particles with a hit quality index (HQI) of <200, or any particles identified as non-plastic material, were excluded from analysis [27].

### 2.4. Statistical Analysis

Mean MP concentrations (MPs/L) were log10-transformed prior to analysis to reduce skewness and stabilize variance. Statistical analyses focused on the four a priori comparisons defined in the study design: (1) flooded field A vs. flooded field B in 2023, (2) flooded field A in 2023 vs. 2024, (3) TWRP before vs. after, and (4) TWRD before vs. after. Because each comparison involved only two groups with limited sample sizes, separate linear models were fitted for each planned contrast, with log-transformed MP concentration as the response variable and the relevant grouping factor (Site, Year, or Treatment) included as the fixed effect. Specific hypotheses for each contrast were evaluated using estimated marginal means and pairwise comparisons. These contrasts tested whether MP concentrations differed between fields with different flooding durations (fall only vs. fall–winter), between years within the same field, and before versus after flooding within the tailwater recovery system. Type II sums of squares were used to obtain F-statistics for fixed effects via the car package and pairwise contrasts were computed using the emmeans package [32,33]. A *p*-value < 0.05 was considered statistically significant in all analyses. All statistical analyses were conducted in R version 4.4.2.

## 3. Results and Discussion

### 3.1. Average Microplastic Concentrations and Common Polymers

Both fields were flooded using the same PE piping and are managed by the same farmer. Any anthropogenic sources of MPs are likely relatively the same since the same machinery, tillage practices, and flooding methods were utilized for both locations. Fields are only 350 m apart, subjecting them to similar exposure levels of MPs from rainfall and atmospheric deposition. Field B was flooded through the winter, whereas Field A was only flooded in the fall. Despite this variation in flooding regime, mean MP concentrations were not statistically significantly different (Table 1). Additionally, mean MP concentrations and polymer types were compared between field A floodwater from Fall 2023 and 2024 (Table 1).

Log-transformed MP concentrations did not differ significantly between flooded field A and flooded field B in 2023 (F_1,10_ = 1.84, *p* = 0.205). Similarly, no difference was detected between years within field A, with MP concentrations in 2023 and 2024 being nearly identical (F_1,10_ < 0.01, *p* = 0.989). Within the tailwater recovery system, MP concentrations tended to decrease after flooding in both TWRP and TWRD; however, these differences were not statistically significant. For TWRP, the post-flood group showed lower MP concentrations than the pre-flood group (estimate = −0.666), but the contrast was not significant (F_1,2_ = 8.02, *p* = 0.105). A similar decreasing pattern was observed in TWRD (estimate = −0.948), although the contrast was also not significant (F_1,1_ = 17.28, *p* = 0.150). These results suggest a consistent directional pattern, although the available sample sizes limit the statistical strength of the evidence.

Following the flood event, both the TWRP and TWRD sites exhibited a distinct downward trend in MP concentrations. Although the effect sizes of these changes (–0.666 and –0.948 log units for TWRP and TWRD, respectively) were substantial relative to within-group variability, these differences did not reach statistical significance. This outcome likely stems from the extremely small sample sizes in the pre-flood and post-flood groups (2 vs. 2 for TWRP; 1 vs. 2 for TWRD), which not only limited statistical results but also precluded a reliable estimation of inter-group differences. Nevertheless, given the consistent direction of change observed at both sites, these findings suggest that flood events may indeed lead to a reduction in MP concentrations within tailwater recovery systems—a phenomenon that warrants further in-depth investigation in future studies through the use of larger sample sizes.

**Figure 2:**
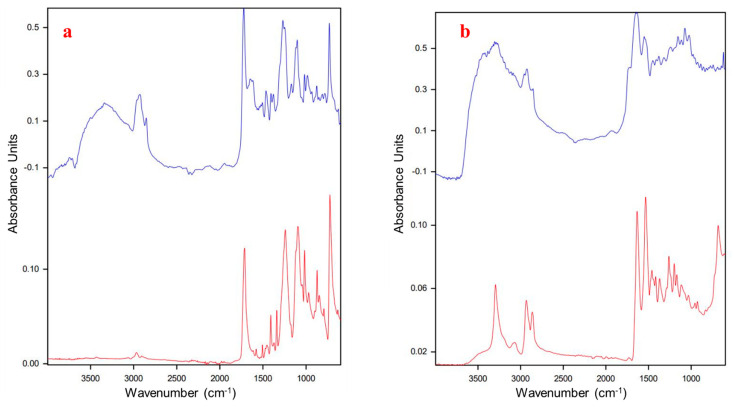
FTIR spectra of (**a**) PET standard (red, **bottom**) with HQI of 976, compared to a microplastic from the agricultural field (sample) spectrum identified as PET (blue, **top**) with HQI of 552. Note the hydroxyl band at ~3300 wavenumbers for the weathered (oxidized) microplastic. (**b**) PA standard (red, **bottom**) with HQI of 904, compared to a microplastic from the agricultural field (sample) spectrum identified as PA (blue, **top**) with HQI of 401.

All identified MPs were matched to database spectra for identification. After identification, the HQI is recorded, and the spectra can be overlayed. The HQI is a more efficient indicator of the spectral match since visual comparison can become difficult. The following FTIR spectra (Figure 2) demonstrate the ability to match up major peaks, but defining how well this spectrum fits based on visuals alone is not possible.

Previous field parameters recorded by those studying shallow water habitat management practices for migratory birds reported that these fields have fine, clayey soil that runs very deep [23]. In addition to this soil description, fields were observed to have a 0–0.5% slope [23]. These parameters suggest a high capacity to retain flood and irrigation water, as well as any contaminants. However, oversaturating the field soil will result in runoff into the TWRD. Therefore, it is important to analyze MP contamination in both the TWRP and TWRD to determine how flooding contributes to the overall transport of MPs.

There was not a significant difference in the mean concentrations of the TWRP water samples before and after flooding, although there appears to be a large decrease in the number of particles. Only two samples were collected before and after flooding, so further sampling is necessary to determine if any relationships exist due to flooding. A decrease in the number of MPs in the TWRP after flooding suggests sources of plastics were present and utilized in the field, warranting further investigation of the TWRD.

Analyzing irrigation water and runoff for this system was important because tailwater recovery systems use recycled water. If high concentrations of MPs in the TWRP are transported, field soils could serve as a sink for MPs. However, if the MP concentrations of the TWRD are elevated, flooding may dislodge MPs from the soil, transporting them in the floodwater and into runoff.

MP concentrations were much lower in the TWRD as compared to the TWRP. Once again, there are not enough samples to establish a significant relationship between the mean concentrations before and after flooding, and further sampling is necessary. However, the lower means in the TWRD as compared to the TWRP suggest that plastics utilized in the agricultural production process have degraded to MPs, but they are not being flushed into runoff. Flooding does not seem to dislodge a significant number of MPs from the soil; instead, MPs formed from plastic materials enter the field where they likely remain floating until they sink into the soil and become lodged. While this study cannot fully explain the transport mechanisms of MPs in detail, MP concentrations in source water and runoff reveal the fate of MPs in floodwater is likely in the soil, not in runoff.

In the edge-of-field runoff samples, the two fields’ mean concentrations were not significantly different for any of the 12 measured runoff events (Table 1). For all runoff samples, mean concentrations were 88 ± 76 MPs/L. The mean ±SE concentration in floodwater samples taken from fields A and B (*n* = 18) was 72 ± 66 MPs/L. These data suggest that MP concentrations are relatively consistent throughout rainfall events that induce edge-of-field runoff. Additionally, they suggest there is relatively no difference in MP concentrations of floodwater and runoff water.

In addition to mean MP concentrations, polymer IDs must also be considered to determine if the varieties of contaminants are relatively similar. Both fields had wide varieties of polymers (Table 1); however, the most prevalent types in both fields were PET, PE, PE/EVA, and TPEs.

Similar to mean MP concentrations between fields A and B, the common polymers present in floodwater are relatively the same. Farming practices and similar exposure to rain and atmospheric deposition of MPs subject these fields to the same kinds of polymers. Polymers present in high abundance in Field B, such as PF, may be a result of a longer flood time. Floodwater applied in December and January could have resulted in higher exposure to PF as compared to Field A where no additional floodwater was applied. Overall, however, the most prominent polymers were present in both fields.

Additionally, polymer types found in field A were compared between floodwater samples from 2023 and 2024 (Table 1). There was a wide variety of polymers, but the most prevalent were once again PET, PE, PE/EVA, and TPEs.

Similarity in polymer distributions between years suggests the same polymers are impacting these agricultural sites from year to year. Since the same management practices and environment remain relatively constant from year to year, sources of MPs are also constant. These similarities highlight that µ-FTIR analysis is an important method for identifying and quantifying MPs in environmental water samples, as concentration data alone may hide shifts in the composition of the MPs present.

To understand which MPs are most commonly found in the field, all floodwater field sample data were combined. The most prevalent polymers, PE, PE/EVA, and PET, are all listed as some of the most common agricultural plastics reported in the United Nation’s assessment of agricultural plastics [6]. Polyethylene is most commonly used in rigid protective products or films such as mulching and bale films. Cotton harvested from fields is rolled into bales with a film, and several cotton farms are adjacent to the sampling locations. With these films in such proximity, photodegradation and transport via wind could deliver MPs to Fields A and B. Additionally, EVA is listed as a common copolymer in film materials that are soft, flexible, and rubberlike. Polyethylene terephthalate is common in plastic bottles housing liquids, so any pesticide containers could be sources of PET contamination. While this study was not able to perform direct source matching with all the MPs present, nearby potential MP sources warrant further investigation into how these widespread plastic products contribute to MP pollution in agricultural environments.

In addition to the floodwater, source water and runoff were analyzed for polymer distributions before and after flooding (Table 1). This provides a general understanding of which types of polymers are being transported most often. Low abundance of PE in source water prior to flooding suggests that the high PE abundance in the field is due to the PE irrigation tubing used to flood the field, previous PE contamination in the soil, or other external sources of PE. Once in the field, these MPs may remain suspended in floodwater or become lodged in the soil. To determine the fate of MPs in this system, the runoff from the TWRD was also collected. Polymer distributions from the TWRD pre-flood are similar to the pre-flood TWRP; however, they differ after flooding occurs. Polyamide, PTFE, and ABS are present in the post-flood TWRD but were not common polymers in floodwater samples. As a result, it is likely that the most prevalent particles remain on the field suspended in floodwater until they settle onto the soil.

At the second site (C, D) in the northern Mississippi Delta, similar polymers were identified. Both PET and PE/EVA were observed in the floodwater collected from the first site, and they occurred in high abundance in runoff from the second site as well. However, new polymers such as polyester (PEST) occur in higher abundance in the floodwater, indicating a slight variation in contaminants. Despite these differences, the most common polymers in both floodwater and runoff seem to be consistent, even from different sites within the Mississippi Delta.

### 3.2. Comparison to Other Agricultural Studies

Concentrations of these polymers in the current study are similar to those previously reported (Table A1). Most studies report MP concentrations in water of around 0–30 MPs/L, while some report concentrations from 100–1000 MPs/L. The current study and another study in Sunflower County, MS, confirm MP pollution in these agricultural waters [11]. The region’s history of intensive agricultural activity may be exposing it to higher levels of MP pollution. The extreme variation in concentrations between agricultural studies makes it difficult to compare the results. Each field and region is subjected to vastly different conditions and levels of plastic contamination; however, further monitoring and identification of plastic sources may help provide a baseline for comparisons.

### 3.3. Microplastic Size Distributions

**Figure 3:**
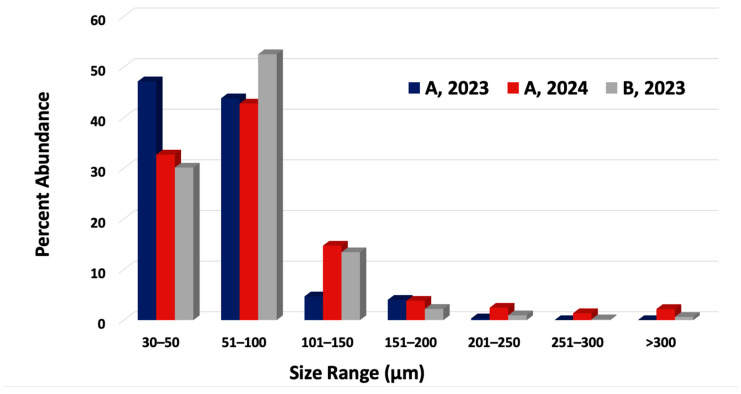
MP Size Distributions in floodwater from Fields A (2023, 2024) and B (2023).

In both fields and during both years, MP sizes were small, with most particles 30–100 µm (Figure 3). Microplastics in the smallest fraction accounted for 91% of MPs in Field A in 2023, followed by 82.6% in Field B in 2023, and 75.5% in Field A in 2024. Microplastics measuring less than 30 µm were not quantified, as the Monel screen used in the one-pot preparation method has a pore size of 30 µm. Smaller MP sizes may result from their ability to be dislodged from soil more easily than MPs of a greater size [16].

The prevalence of smaller MPs is concerning due to their continued exposure to UV light and potential for continued photodegradation and fragmentation. Smaller MPs and nanoplastics have been found to disrupt soil microbial communities and interfere with plant roots [34,35]. Additionally, their extremely small size makes filtration and remediation even more difficult. Some current remediation strategies include heat treatment of biosolids/sludge (hyperthermophilic composting), biodegradation by periphytic biofilms, as well as filtration with biochar [12,36,37]. Remediation techniques have not been standardized for agricultural systems, and the efficacy will likely vary with MP size and contamination level. Biochar has demonstrated a preferential ability to trap smaller tire wear particles compared to larger sizes, particularly under specific environmental or experimental conditions [11].

**Figure 4:**
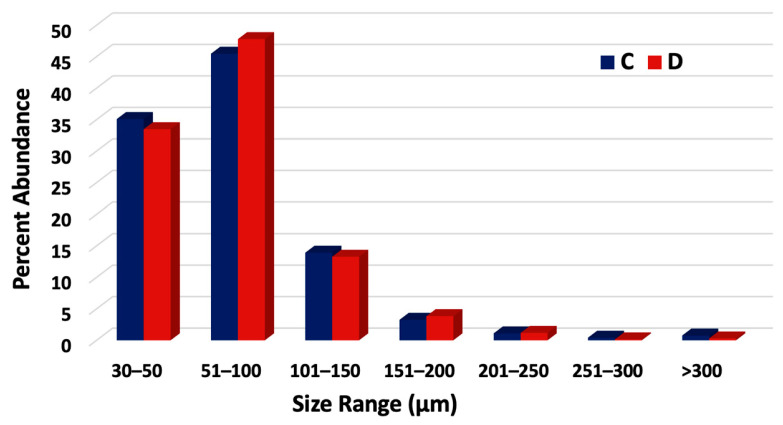
MP Size Distributions in runoff from Fields C and D during rain events between September 2024 and February 2025.

Trends similar to those at sites A and B were also observed in runoff samples from sites C and D (Figure 4). Most particles (80.8%) measured between 30–100 microns. In both stagnant floodwater and edge-of-field runoff, most MPs were in the smallest size fractions measured.

### 3.4. Source Identification of Plastic Litter

Ten pieces of plastic litter were collected from fields A and B. Although the items cannot be directly matched to MPs present in the previous analyses, they demonstrate a small variety of plastic pollution present in and around the field of study. Samples fall into two categories: those related to agricultural activity (irrigation pipe, mulching film, tarps) and those from other sources, such as human activity (drink bottles, shotgun shells).

Half of the plastic litter was identified as PE, including irrigation polypipe used to flood the field (Table 2). This piping was laid out along the eastern border of the field, exposing it to sunlight. Degradation of this tubing could subject the field to MPs, and there was a higher relative abundance of PE MPs identified in floodwater samples. However, samples such as oil and milk bottles were also identified as PE, so PE contamination cannot be attributed to agricultural materials alone. This same trend occurs for other polymers such as PET and PP, which are used in manufacturing agricultural materials as well as drink bottles found in the fields. Polymer IDs with an HQI < 700 (Table 2) suggest that MPs have been subjected to some level of aging and weathering, resulting in slight changes to their surface properties compared to the other MPs. As MPs are exposed to environmental conditions over time, their physicochemical properties evolve, altering their structural properties. This explains the lower HQI values observed, in agreement with previous findings [38].

## 4. Conclusions

Agricultural waters are an important area of study for MP contamination, especially given the wide use of plastic polymers in the field. Irrigation water, floodwater, and runoff have all been shown to contribute to MP contamination, posing a potential threat to agricultural systems. This study was intended to characterize the concentrations, polymers, and sizes of MPs present in both post-harvest (intentionally) flooded fields and in edge-of-field runoff in the Mississippi Delta. The most common polymers identified were PET, PE, PE/EVA, and TPEs, all of which are some of the most used plastics in agriculture. Floodwater concentrations (*n* = 18) averaged 72 ± 66 MPs/L and runoff concentrations (*n* = 12) averaged 88 ± 76 MPs/L, which are slightly elevated compared to other studies. Results are indicative of a potential issue with plasticulture’s role in contaminating agricultural environments with emerging contaminants still under review for their impacts on human health. While this study identifies the most prevalent polymers, a primary limitation remains the inability to definitively link these materials to specific agricultural practices. Further analysis is required to differentiate between primary sources, such as irrigation materials and films, as well as other external pathways of contamination. This work should focus on the degradation of agricultural plastics over time in comparison to other plastic sources, which includes analyzing common morphologies, surface structure modifications, and spectral changes.

Results also demonstrate that MPs are being distributed on the field via floodwater. After flooding, lower MP concentrations in runoff indicate that MPs become lodged in the soil, which is likely due to the minimal field slope and clayey soil type. Moreover, MP sizes are relatively small, with most particles (75.5–91%) occurring in the smallest size fraction measured (30–100 µm). It is important to note that this study is limited to particles 30 µm and greater, given that any particles below this threshold would pass through the Monel mesh screen used in sample processing. Future work is required to fully understand the soil MP concentrations, and ultimately the fate of MPs in this agricultural system as well as similar flooded field systems.

In addition to flooding fields for improving soil quality and creating habitats for migratory shorebirds post-harvest, flooding is commonly used in rice cultivation. Flooding rice fields irrigates the crop and reduces weed and insect pressures. Some farmers use multiple-inlet irrigation, which relies on plastic piping [39]. The piping is laid out perpendicular to levees so that all paddies are irrigated simultaneously [39]. In other fields, farmers opt for plastic film mulching combined with drip irrigation, which covers each row of crops in a layer of plastic before flooding [40]. Both irrigation methods are likely to expose rice paddies to MPs because materials are laid out in direct exposure to UV light, and the floodwater can float and transport MPs around the field. Future work is needed to understand how plastic materials are impacting rice irrigation water, and if MPs are being transported into runoff or into the roots of rice varieties themselves.

## Figures and Tables

**Table 1:** Mean Microplastic Concentrations and Common Polymers at All Sites.

Site Name	*n*	Mean Concentration (MPs/L)	Standard Error	Common Polymers
A, 2023	6	50	13	PET (19%), TPEs (15%), PE (14%)
A, 2024	6	61	19	PET (17%), PBT (12%), PE (11%)
B, 2023	6	105	40	PF (15%), PET (15%), PE/EVA (14%)
TWRP Before	2	271	13	PF (21%), PA (18%), PET (13%), TPEs (12%)
TWRP After	2	67	33	PE/EVA (29%), PA (19%), PET (14%)
TWRD Before	1	72	-----	PET (31%), PE/EVA (24%), PF (15%)
TWRD After	2	9	3	PTFE (18%), PA (17%), ABS (12%)
C	12	120	25	PET (34%), PE/EVA (9%)
D	12	57	13	PET (27%), PBT (10%)

PET = polyethylene terephthalate; TPEs = thermoplastic elastomers; PE = polyethylene; PF = phenyl formaldehyde; PE/EVA = polyethylene/ethyl vinyl acetate co-polymer; PA = Polyamide; PTFE = polytetrafluoroethylene; ABS = acrylonitrile butadiene styrene; PBT = polybutylene terephthalate.

**Table 2:** Polymer IDs, Percent Abundance, and Hit Quality Indices for Collected Plastic Litter.

Sample Description	Polymer ID	Percent Abundance	HQI
Blue zip tie	Polyamide	10	526
Plastic irrigation pipe	Polyethylene		786
Black mulching film			720
Yellow shotgun shell		50	756
White oil bottle			674
Milk bottle			907
Clear soda bottle	Polyethylene terephthalate	20	983
Unknown drink bottle			488
Soda bottle label	Polypropylene	10	909
Blue tarp strand	Polypropylene/thermoplastic polyolefin	10	741

## Data Availability

The original contributions presented in this study are included in the article. Further inquiries can be directed to the corresponding author.

## Abbreviations

The following abbreviations are used in this manuscript:

ABS	acrylonitrile butadiene styrene
ATR	attenuated total reflectance
CM	chlorinated polyethylene
EVA	ethyl vinyl acetate
FEP	fluorinated ethylene propylene
FTIR	Fourier transform infrared spectroscopy
GI	gastrointestinal
HQI	hit quality index
IR	infrared
MP	microplastic
NP	nanoplastic
PA	polyamide, “nylon”
PARA	polyarylamide
PBT	polybutylene terephthalate
PC	polycarbonate
PCBs	polychlorinated biphenyls
PE	polyethylene
PET	polyethylene terephthalate
PF	phenyl formaldehyde
PMMA	poly(methyl methacrylate)
PP	polypropylene
PS	polystyrene
PTFE	polytetrafluoroethylene, “teflon”
PU	polyurethane
PVC	polyvinyl chloride
Pyr-GC-MS	pyrolysis gas chromatography mass spectrometry
TPEs	thermoplastic elastomers
TPO	thermoplastic polyolefin
TPU	thermoplastic polyurethane
TWRD	tailwater recovery ditch
TWRP	tailwater recovery pond

## Appendix A

**Table A1:** Microplastic Concentrations, Common Polymers, and Sizes from Other Agricultural Studies.

Author/Study	Location	Year	Crop(s)	Sample Type	Most Common Polymers	MP Concentrations	Common MP Sizes (µm)	Potential Sources
This Study	Indianola, MS, USA	2023	Corn, soybeans	Floodwater	PET, PE/EVA, PE/EVA	50 and 105 MPs/L on average in two fields	30–100	PE irrigation piping, litter around field
		2024				61 MPs/L on average	30–100	
Olubusoye et al. [11].	Indianola, MS, USA	2024	Corn, soybeans, cotton	Field runoff	PE, PA, PVC, PU	27–609 MPs/L, average 279	-----	Sewage sludge, atmospheric deposition, PE sheeting
Liu et al. [15].	Bishan District, Chongqing Municipality City, China	2024	Cabbage	Field runoff	PE, CPE, PU	146–2043 MPs/L	20–50	Fertilizers
				Soil	PE, PP	40,614–52,676 MPs/kg soil	<50	
Lwanga et al. [41].	Netherlands	2023	Sugar beets, carrots, asparagus, pumpkins, apples, pears	Water	LDPE, PAC, PE, PP	11 +/− 4.7 MPs/L	50–150	Plastic mulching
				Soil		67–1109 MPs/g soil	50–150	
Beni et al. [42].	Lincoln, NE, USA	2023	Corn, soybeans	Field runoff	PE, PET, PP	10–31 MPs/L	-----	Biosolids, manure
Bigalke et al. [43].	Seeland District, Switzerland	2022	Various vegetables	Field runoff	PA and PE	10.5 +/− 9.5 MPs/L	100–150 (lower sizes not quantified)	Mulch, greenhouse, and tunnel foils
Schell et al. [44].	Madrid, Spain	2022	Barley	Field runoff	Pl, PP, PEST	0–14 MPs/L	-----	Sewage sludge
				Soil	PEST and acrylic	Averages of 330, 204, 215, 211 MPs/g soil in various fields	-----	Irrigation water, sludge, and greenhouse films
Grbić et al. [45].	Lake Ontario, Canada	2020	-----	Field runoff, downstream	PVC, PP	0.9 +/− 1.3 MPs/L	-----	Irrigation water, sludge, and greenhouse films
